# Ion channel chameleons: Switching ion selectivity by alternative splicing

**DOI:** 10.1016/j.jbc.2023.102946

**Published:** 2023-01-24

**Authors:** Allen L. Hsu, Manu Ben-Johny

**Affiliations:** Department of Physiology and Cellular Biophysics, Columbia University, New York, New York, USA

**Keywords:** ion channels, calcium channels, ion selectivity, CaV3, lymnaea CaV3, T-type channels, sodium channels, AP, action potential, CaV, Voltage-gated calcium channel, LCaV3, Lymnaea CaV3, NaV, Voltage-gated sodium channel, SF, selectivity filter

## Abstract

Voltage-gated sodium and calcium channels are distinct, evolutionarily related ion channels that achieve remarkable ion selectivity despite sharing an overall similar structure. Classical studies have shown that ion selectivity is determined by specific binding of ions to the channel pore, enabled by signature amino acid sequences within the selectivity filter (SF). By studying ancestral channels in the pond snail (*Lymnaea stagnalis*), Guan *et al.* showed in a recent JBC article that this well-established mechanism can be tuned by alternative splicing, allowing a single Ca_V_3 gene to encode both a Ca^2+^-permeable and an Na^+^-permeable channel depending on the cellular context. These findings shed light on mechanisms that tune ion selectivity in physiology and on the evolutionary basis of ion selectivity.

Voltage-gated sodium (Na_V_) and calcium channels (Ca_V_) are members of an evolutionarily related ion channel superfamily that function in essential physiological processes. The Na_V_ channels are responsible for initiation and propagation of action potentials (APs) in excitable cells, while Ca_V_ channels transduce voltage changes to the influx of Ca^2+^ ions, a second messenger process that tunes excitability and supports synaptic neurotransmission, excitation-contraction coupling, and gene regulation (1). In mammals, these channels are multisubunit complexes encoded by distinct genes. At the molecular level, the pore-forming subunit of the two channel families share a similar pseudo-tetrameric architecture with four homologous domains linked together by intracellular segments. Each domain contains a voltage-sensing domain formed by four transmembrane helices (S1–S4) and two helices (S5–S6) that line the central ion permeation pathway or the channel pore. The pore contains an SF that determines ion selectivity. Given this broad similarity, understanding structural and molecular mechanisms that confer ion selectivity is of fundamental biological importance.

From an evolutionary perspective, Ca_V_ channels are thought to have emerged and diversified in early unicellular eukaryotes in conjunction with the increased importance of sophisticated Ca^2+^ signaling mechanisms (2). By comparison, eukaryotic Na_V_ channels are thought to have evolved from Ca_V_ channels in early metazoans, predating the nervous system (3). Paradoxically, some animals appear to lack homologous genes encoding Na_V_ channels, yet they exhibit voltage-dependent Na^+^ current essential for AP generation (4). In a recent issue of the Journal of Biological Chemistry, Guan *et al.* studied ion channels in the heart of the giant pond snail (*L. stagnalis*), where APs are driven by both Na^+^ and Ca^2+^ currents, yet only a single Ca_V_ gene appears to be expressed. They uncovered an intriguing mechanism involving alternative splicing of a Ca_V_3 gene, a homolog of the mammalian low voltage–activated T-type channel, which switches its preference from Ca^2+^ to Na^+^ ions. The authors leveraged emerging deep learning-based structure prediction approaches and electrophysiology to identify the molecular basis of this process (5).

Extensive study over the past 50 years has identified core features responsible for the ion selectivity of voltage-gated ion channels. Classical biophysical studies point to a scheme where the pore domain contains two or more high affinity–binding sites for the specific ion (6). Once bound, coulombic interactions between pairs of ions permit robust flux of the ion despite high affinity binding. These expectations were largely substantiated by structural studies, which revealed the atomic arrangement of the SF (7) formed by two pore helices (P1–P2). This results in a channel pore with specific dimensions and distinct amino acid sequences that line the ion permeation pathway. For Ca_V_ channels, the signature sequence is a ring of four negatively charged residues, typically glutamates (‘EEEE’), that line the narrow SF (6). Additionally, a closely juxtaposed aspartate residue (D[+1], indicating its location immediately C-terminal to the central glutamate) in domain II (DII) is also important for Ca^2+^ coordination (8). For Na_V_ channels, the SF contains an aspartate-glutamate-lysine-alanine or ‘DEKA’ sequence. The additional aspartate residue is typically absent. Like other Ca^2+^ channels, the *Lymnaea* Ca_V_3 channel contains a ring of negatively charged residues (‘EEDD’) and D[+1] residue (4). So how is this *bona fide* Ca^2+^ channel able to masquerade as an Na^+^ channel?

The Spafford lab has previously shown that the *Lymnaea* Ca_V_3 (LCa_V_3) channels are alternatively spliced, resulting in distinct variants that are expressed in a cell type–dependent manner (Fig. 1) (4). In the snail heart, the predominant LCa_V_3 channel isoform includes exon12a. Inclusion of this exon increases Na^+^ permeability. By contrast, the skeletal muscle variant of LCa_V_3 includes exon12b, which increases Ca^2+^ permeability (4). In their recent work, the authors integrated computational structural prediction using AlphaFold with electrophysiological analysis to determine how structural changes outside of the classical SF can impact ion permeation. In the predicted Ca_V_3 models from *L. stagnalis*, the exon12a channel possesses a lysine residue (K[1067]), which is hypothesized to form a salt bridge with the D[+1] residue (Fig. 1). In channel variants with exon12b, an alanine (A[1078]) is present in place of the lysine and is incapable of forming a salt bridge (Fig. 1). Guan *et al.* propose that the presence of the lysine residue neutralizes the DII D[+1] residue and change the overall local charge density in the outer pore vestibule. When the D[+1] residue is present (*e.g.*, with exon12a), the Ca^2+^ ion is coordinated by the carboxylate groups of the outer D[+1] and the SF aspartate/glutamates. When an Na^+^ ion enters the pore, it is repelled by this Ca^2+^, while incoming Ca^2+^ ions knock off the chelated Ca^2+^ ion into the inner pore of the channel. With the D[+1] charge neutralized, channels then allow influx of Na^+^ ions.

**Figure 1:**
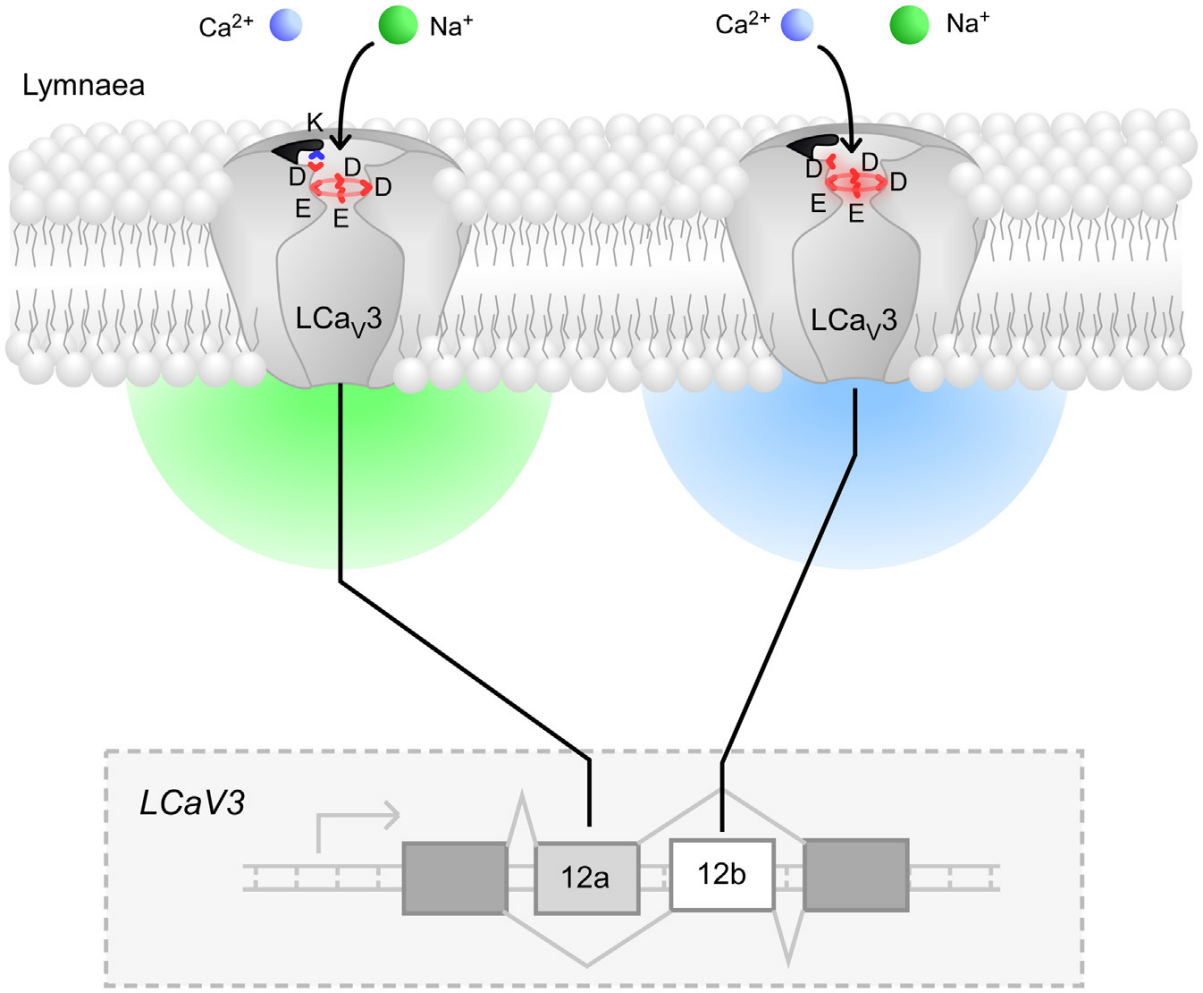
**Alternative splicing of *Lymnaea stagnalis* Ca**_**V**_**3 channel switches ion selectivity.** Exon 12a contains a positively charged lysine residue that neutralizes an Aspartate residue critical for Ca^2+^ permeability, thus allowing Na^+^ influx through a *bona fide* Ca^2+^ channel.

To test their hypothesis, Guan *et al.* mutated the key lysine residue in the LCa_V_3 exon12a variant into alanine and estimated changes in Na^+^ permeability. Indeed, the peak Na^+^ current size with the LCa_V_3-exon12a K[1067]A mutant was significantly reduced in comparison to the WT LCa_V_3-exon12a variant, indicating reduced Na^+^ permeability. Conversely, replacement of the A[1078] residue of LCa_V_3-exon12b with a lysine led to increased Na^+^ currents.

Overall, this study provides new insights into both biophysical and evolutionary mechanisms that determine ion selectivity for voltage-gated channels, a long-standing physiological problem. In mammals, alternative splicing of Ca^2+^ channels is increasingly recognized as a versatile mechanism for fine tuning various channel properties including subcellular localization and gating. The present study illustrates that splicing can also impact Ca^2+^ permeability, effectively allowing a Ca^2+^ channel to become a *de facto* Na^+^ channel. What remains unknown, however, is the physiological and evolutionary benefit of this atypical splicing-dependent mechanism, in place of a dedicated Na_V_ gene. It is possible that alternative splicing may be further regulated either developmentally or in response to other stimuli. Another possibility is that the Na^+^ *versus* Ca^2+^ permeability of LCa_V_3 may have distinct effects on shaping APs *versus* tuning excitation-contraction coupling in the *Lymnaea* heart. Biophysically, the finding that domains outside of the ion permeation pathway can tune the channel selectivity has important implications. A dizzying array of genetic mutations in nearly all Ca_V_ and Na_V_ channels have been linked to a wide range of human diseases; it is possible that some of these mutations may impact channel selectivity, leading to an imbalance in ion homeostasis. Excitingly, this study also highlights the potential utility of deep learning-based computational structure prediction approaches to generate new hypotheses to address long-standing physiological problems (9). Combining *in silico* predictions with carefully designed electrophysiological studies could substantially aid structure-function analysis of ion channel regulation. Even still, experimental structural determination using, for example, cryo-EM will be essential to fully substantiate possible structural mechanisms.

## Conflict of interest

The authors declare that they have no conflicts of interest with the contents of this article.
